# Positive horizontal-canal head impulse test is not a benign sign for acute vestibular syndrome with hearing loss

**DOI:** 10.3389/fneur.2022.941909

**Published:** 2022-09-26

**Authors:** Anand K. Bery, Tzu-Pu Chang

**Affiliations:** ^1^Division of Neurology, Department of Medicine, University of Ottawa, Ottawa, ON, Canada; ^2^Department of Neurology/Neuro-Medical Scientific Center, Taichung Tzu Chi Hospital, Buddhist Tzu Chi Medical Foundation, Taichung, Taiwan; ^3^Department of Neurology, School of Medicine, Tzu Chi University, Hualien, Taiwan

**Keywords:** vertigo, dizziness, acute vestibular syndrome, hearing loss, central vestibulopathy, stroke, head impulse test, nystagmus

## Abstract

**Background:**

Diagnosis of acute vestibular syndrome (AVS) with hearing loss is challenging because the leading vascular cause—AICA territory stroke—can appear benign on head impulse testing. We evaluated the diagnostic utility of various bedside oculomotor tests to discriminate imaging-positive and imaging-negative cases of AVS plus hearing loss.

**Method:**

We reviewed 13 consecutive inpatients with AVS and acute unilateral hearing loss. We compared neurologic findings, bedside and video head impulse testing (bHIT, vHIT), and other vestibular signs (including nystagmus, skew deviation, and positional testing) between MRI+ and MRI– cases.

**Results:**

Five of thirteen patients had a lateral pontine lesion (i.e., MRI+); eight did not (i.e., MRI–). Horizontal-canal head impulse test showed ipsilateral vestibular loss in all five MRI+ patients but only in three MRI– patients. The ipsilesional VOR gains of horizontal-canal vHIT were significantly lower in the MRI+ than the MRI– group (0.56 ± 0.11 vs. 0.87 ± 0.24, *p* = 0.03). All 5 MRI+ patients had horizontal spontaneous nystagmus beating away from the lesion (5/5). One patient (1/5) had direction-changing nystagmus with gaze. Two had skew deviation (2/5). Among the 8 MRI– patients, one (1/8) presented as unilateral vestibulopathy, four (4/8) had positional nystagmus and three (3/8) had isolated posterior canal hypofunction.

**Conclusion:**

The horizontal-canal head impulse test poorly discriminates central and peripheral lesions when hearing loss accompanies AVS. Paradoxically, a lateral pontine lesion usually mimics unilateral peripheral vestibulopathy. By contrast, patients with peripheral lesions usually present with positional nystagmus or isolated posterior canal impairment, risking misdiagnosis as central vestibulopathy.

## Introduction

Acute vestibular syndrome (AVS) is defined as prolonged vertigo, vomiting, or unsteadiness more than 24 h (1, 2). The most common cause of AVS is vestibular neuritis (representing ~75% of AVS), followed by posterior fossa stroke (~25% of AVS) (3, 4). Differentiating central from peripheral causes of AVS remains a diagnostic challenge. The bedside oculomotor exam battery known as HINTS (i.e., Head Impulse test, Nystagmus, and Test of Skew deviation), is an accurate method for clinically distinguishing stroke from AVS (5). HINTS is designed to maximize sensitivity for detecting stroke; thus, to conclude a peripheral cause for vertigo, each of the three bedside maneuvers must point to a peripheral etiology. In other words, one requires positive head impulse test (HIT) AND unidirectional horizontal nystagmus AND negative test of skew to conclude a peripheral etiology. Any ONE of the following: negative HIT, direction-changing nystagmus, positive test of skew suggests that a central lesion is possible. Among the three tests, a positive HIT (6) (where an ocular refixation is seen) suggests a peripheral etiology since it usually indicates vestibular neuritis while normal HIT could portend a stroke (3, 5).

Stroke in the territory of AICA (the anterior inferior cerebellar artery) presents particular challenge because it is a central event (stroke) but can present with a positive HIT, which may be incorrectly interpreted as a benign finding. Because the blood supply of the inner ear originates from AICA, AICA infarction is often accompanied by labyrinthine ischemia and presents as acute audio-vestibular loss (7, 8). Therefore, these patients experience acute unilateral hearing loss with a positive HIT on the side of hearing loss, and are hard to differentiate from those with pure peripheral audio-vestibular loss. In order not to miss this type of stroke, the addition of acute hearing loss to the HINTS battery has previously been proposed (a strategy previously termed “HINTS Plus” for “HINTS Plus hearing loss”) (9). Under HINTS Plus, acute hearing loss is a red flag feature supporting a stroke survey including brain MRI, even if all other signs point to a peripheral disorder.

HINTS Plus remains controversial, in part because infarction is historically felt to be an uncommon cause of sudden sensorineural hearing loss (SSNHL) *without* vertigo. Most cases of SSNHL are believed to be inflammatory rather than vascular in origin, and SSNHL with vertigo has sometimes been termed “labyrinthitis,” which implies viral infection of the labyrinth (10). Some experts feel AICA stroke is a rare cause of SSNHL when compared to viral labyrinthitis (11–13). However, prior epidemiological studies have shown patients with SSNHL have a higher risk of stroke than the general population (14), and SSNHL with vertigo portends greater risk of stroke than either SSNHL or vertigo alone (15).

In this study, we systematically classified a series of patients with AVS plus acute hearing loss into central vs. peripheral etiologies by neuroimaging. We then compared clinical, oculomotor, and vestibular signs among groups to determine which features provide diagnostic value in this challenging clinical presentation.

## Methods

We reviewed consecutive inpatients hospitalized for acute vertigo at our institution's neurology ward (Taichung Tzu Chi Hospital) between July 2017 and March 2022. We retained only those patients who had acute unilateral hearing loss accompanying their vertigo (in other words, the AVS plus acute unilateral hearing loss). AVS was defined as persistent vertigo and/or unsteadiness ≥24 h. Unilateral hearing loss was defined here as patient complaint of newly developing hearing loss that was confirmed by calibrated finger rub auditory screening test (CALFRAST) in the acute stage of vertigo (16). As per standard of care at our institution, all patients with this presentation are hospitalized for stroke workup and MRI study. We excluded patients who had a history of Ménière's disease, vestibular migraine, or other episodic vestibular syndromes.

Patients underwent bedside oculomotor testing by a single vestibular neurologist (TPC) in the ED before admission. This included the bedside head impulse test (bHIT), assessment of spontaneous and gaze-evoked nystagmus (GEN) with unaided eyes, and test of skew deviation. They also underwent positional testing in the ED, including the bow-and-lean test, Dix-Hallpike test, and supine-roll test, all *via* videonystagmoscopy with fixation block (i.e., without fixation). In addition, all patients underwent general neurological examinations in the emergency department, including comprehensive examination of cranial nerve function, muscle strength, myotatic reflexes and Babinski sign, sensory testing, finger-to-nose test, heel-to-shin test, rapid alternating movement, and assessment of gait.

Once hospitalized, patients underwent video-oculography (VOG) with video head impulse testing (vHIT). Patients routinely underwent horizontal-canal vHIT (H-vHIT), and those with normal H-vHIT results further underwent vertical-canal vHIT. A positive result was defined as vestibulo-ocular reflex (VOR) gain <0.8 in H-vHIT or VOR gain <0.75 in vertical-canal vHIT. The patients also underwent the bucket test for subjective visual vertical (SVV) with four trials (17). The mean SVV deviation of four trials >2.3 degrees was defined as abnormal. All patients underwent brain MRI and pure-tone audiometry during hospitalization. The sequences of brain MRI included DWI, T1WI, T2WI, FLAIR, SWAN, and MRA. For audiometry, pure-tone average (PTA) was defined as the average of hearing thresholds at 500, 1,000, and 2,000 Hz. Normal hearing was defined as thresholds ≤25 dB at all frequencies of the audiogram. All the exams were completed within 1 week of vertigo onset.

Patients were classified by MRI findings into two groups; those with posterior fossa lesions were classified as “MRI+,” and those without posterior fossa lesions were classified as “MRI–.” We compared demographic, bedside oculomotor, VOG, and other neurologic findings between groups. This included bHIT, vHIT, spontaneous nystagmus, GEN, positional nystagmus, skew deviation, SVV, PTA, and presence of vascular risk factors. Analysis was done in SPSS (IBM, v23). Continuous variables were compared *via* Mann-Whitney *U*-test. *p*-value < 0.05 was considered statistically significant.

## Results

**Figure 1:**
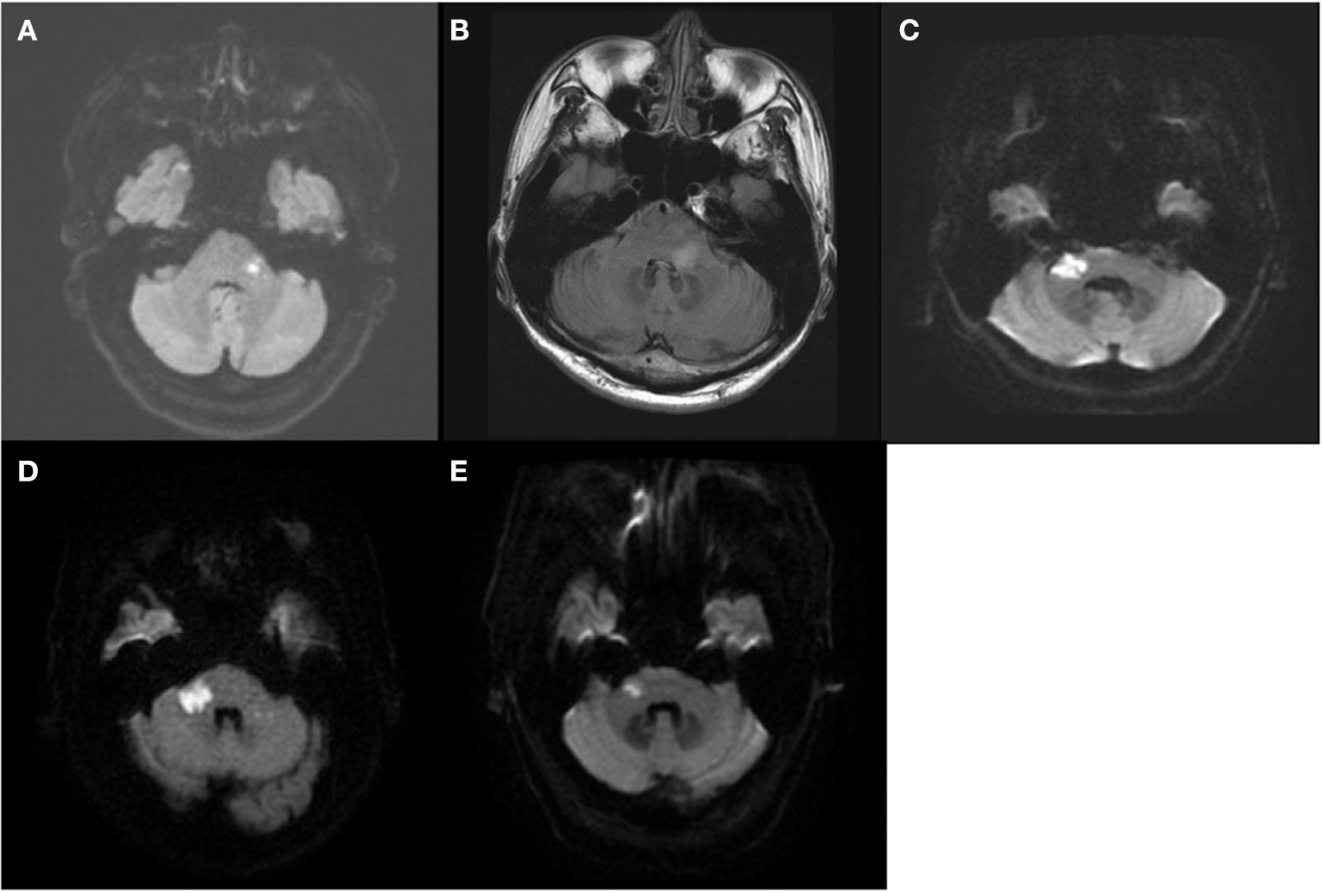
The MRI findings of the five patients with posterior fossa lesion (MRI+ group). Four had acute lateral pontine infarction **(A,C–E)** and one had acute demyelinating lesion in left lateral pons **(B)**.

Thirteen patients with AVS plus acute hearing loss were included. All thirteen patients were hospitalized within 2 days of vertigo onset, and underwent brain MRI between 2 and 5 days (48–120 h) after vertigo onset. Among them, five patients had a posterior fossa lesion on MRI (and thus comprise the “MRI+” group). This includes four patients with acute lateral pontine infarction belonging to AICA territory and one patient with an acute demyelinating lesion in left lateral pons, who was ultimately diagnosed with multiple sclerosis (Figure 1). The other eight patients did not have posterior fossa lesion (and thus comprise the “MRI–” group).

Full clinical and neuro-otologic details of the included patients are available in Table 1. Four patients were female. The mean age was 59.4 years (56.2 in MRI+ and 61.4 in MRI–). Seven patients had diabetes mellitus (2/5 in MRI+ and 5/8 in MRI–), nine had hypertension (4/5 in MRI+ and 5/8 in MRI–), and six had dyslipidemia (4/5 in MRI+ and 2/8 in MRI–). The ABCD2 was not significantly different between groups (mean ABCD2 score 3.6 in MRI+ vs 3.9 in MRI–, *p* = 0.72). Three patients (3/5) in the MRI+ group had focal neurological signs, including one with diplopia and two with a lower motor neuron facial palsy. None of the patients in the MRI– group had focal neurological signs, and none developed focal signs or worsening in gait during hospitalization or follow-up clinic visits at 2 and 4 weeks, respectively, after vertigo onset.

**Table 1:** Clinical and neuro-otologic findings in the patients with acute vestibular syndrome plus hearing loss.

**Pt**	**Age/Sex**	**HL**	**PTA (dB)**	**H-bHIT**	**H-vHIT**		**Fixation**		**Fixation block**		**Skew**	**SVV (°)**	**Other signs**	**MRI**
					**Ipsi-lesional**	**Contra-lesional**	**SN**	**GEN**	**SN**	**PN**				
1	68/F	L	29	L	0.45	1.26	RBN	–	RBN	RBN	–	L, 6	–	–
2	48/M	L	65	L	0.64	1.11	RBN	+	RBN	RBN	+	L, 5	Diplopia	Left lateral pontine infarction
3	41/M	L	25	L	0.56	0.61	RBN	–	RBN	RBN	+	L, 5	Left facial palsy	Left lateral pontine demyelinating lesion
4	46/M	R	49	R	0.49	0.78	LBN	–	LBN	LBN	–	R, 10	–	Right lateral pontine infarction
5	77/M	R	90	R	0.68	0.68	LBN	–	LBN	LBN	–	R, 15	–	Right lateral pontine infarction
6	69/M	R	24	R	0.42	1.17	LBN	–	LBN	LBN	–	R, 1	Right facial palsy	Right lateral pontine infarction
7	62/M	R	90	Normal	1.14	0.92	–	–	–	RBN in right lying	–	L, 1	Right PC impaired at bHIT and vHIT (0.57)^*^	–
8	69/M	R	104	Normal	ND	ND	–	–	LBN	LBN	–	0	Right PC impaired at bHIT	–
9	60/F	R	79	Normal	1.14	1.15	–	–	LBN	LBN	–	R, 2	Right PC impaired at bHIT and vHIT (0.29)^*^	–
10	58/F	R	113	Normal	0.77	0.69	RBN	–	RBN	Geotropic	–	L, 3	–	–
11	62/M	L	115	Normal	0.88	0.88	LBN	–	LBN	Geotropic	–	ND	–	–
12	53/M	R	109	R	0.87	0.86	LBN	–	LBN	Apogeotropic	–	R, 3	–	–
13	59/F	L	79	L	0.82	0.98	RBN	–	RBN	Geotropic	–	L, 1	–	–

HL, hearing loss; PTA, pure-tone average (the average of pure tone thresholds at 500, 1,000, and 2,000 Hz in the lesion ear); H-bHIT, horizontal-canal bedside head impulse test; H-vHIT, horizontal-canal video head impulse test; SN, spontaneous nystagmus; GEN, gaze-evoked nystagmus; PN, positional nystagmus; SVV, subjective visual vertical; F, female; M, male; L, left; R, right; B, bilateral; RBN, right-beating nystagmus; LBN, left-beating nystagmus; PC, posterior semicircular canal; ND, not documented.

* The numbers in the parentheses are the VOR gains of vertical-canal vHIT.

### Bedside head impulse test and vestibular exams

Patients underwent bedside vestibular testing within 2 days of vertigo onset. Horizontal canal bedside head impulse testing (H-bHIT) showed unilateral vestibular loss on the side of hearing loss (i.e., ipsilateral to the lesion side) in all MRI+ patients (5/5). Among MRI+ patients, all (5/5) had horizontal spontaneous nystagmus beating away from lesion side, never changing direction during positional testing (5/5). One MRI+ patient (1/5) had direction-changing gaze-evoked nystagmus (GEN), and two (2/5) had skew deviation.

Among the eight MRI– patients, only three patients (3/8) had unilateral vestibular loss on H-bHIT. One patient (1/8) with positive H-bHIT presented with spontaneous nystagmus beating away from the lesion side which did not change direction during positional testing. Four other patients (4/8, including 2/8 with positive H-bHIT and 2/8 with negative H-bHIT) also had horizontal spontaneous nystagmus *via* unaided eyes, but presented with persistent geotropic (3/8) or apogeotropic nystagmus (1/8) on the supine-roll test. In the other three (3/8) patients with negative H-bHIT, no spontaneous nystagmus could be seen *via* unaided eyes; when observed by video-nystagmoscopy with fixation block, two had weak horizontal nystagmus and one had weak positional nystagmus in right side-lying position. All the directions of nystagmus were mainly horizontal. None of the patients in the MRI– group had GEN or skew deviation (Table 1).

### Video head impulse test

Two to three days after vertigo onset, all but one patient (one MRI– patient with normal H-bHIT refused vHIT) underwent vHIT. Among the MRI+ patients presenting with unilateral vestibular loss in bHIT, two (2/5) had ipsilesional vestibular loss on H-vHIT, and the other three (3/5) had bilateral vestibular loss on vHIT (ipsilesional/contralesional VOR gains were 0.56/0.61 in Pt #3, 0.49/0.78 in Pt #4, and 0.68/0.68 in Pt #5).

On the other hand, only one MRI– patient (1/7) had ipsilesional vestibular loss and one (1/7) had bilateral vestibular loss in H-vHIT. We selectively performed the vertical canal-vHIT for the other five patients having normal H-vHIT to check the function of vertical semicircular canals, and found two patients (2/7) had hypofunction of the posterior canal (PC) in the lesion side. The patient who refused vHIT received vertical-canal bHIT, which also showed PC hypofunction on the lesion side. All three patients with isolated PC hypofunction were those patients whose nystagmus was weak and could only be seen in fixation block.

**Figure 2:**
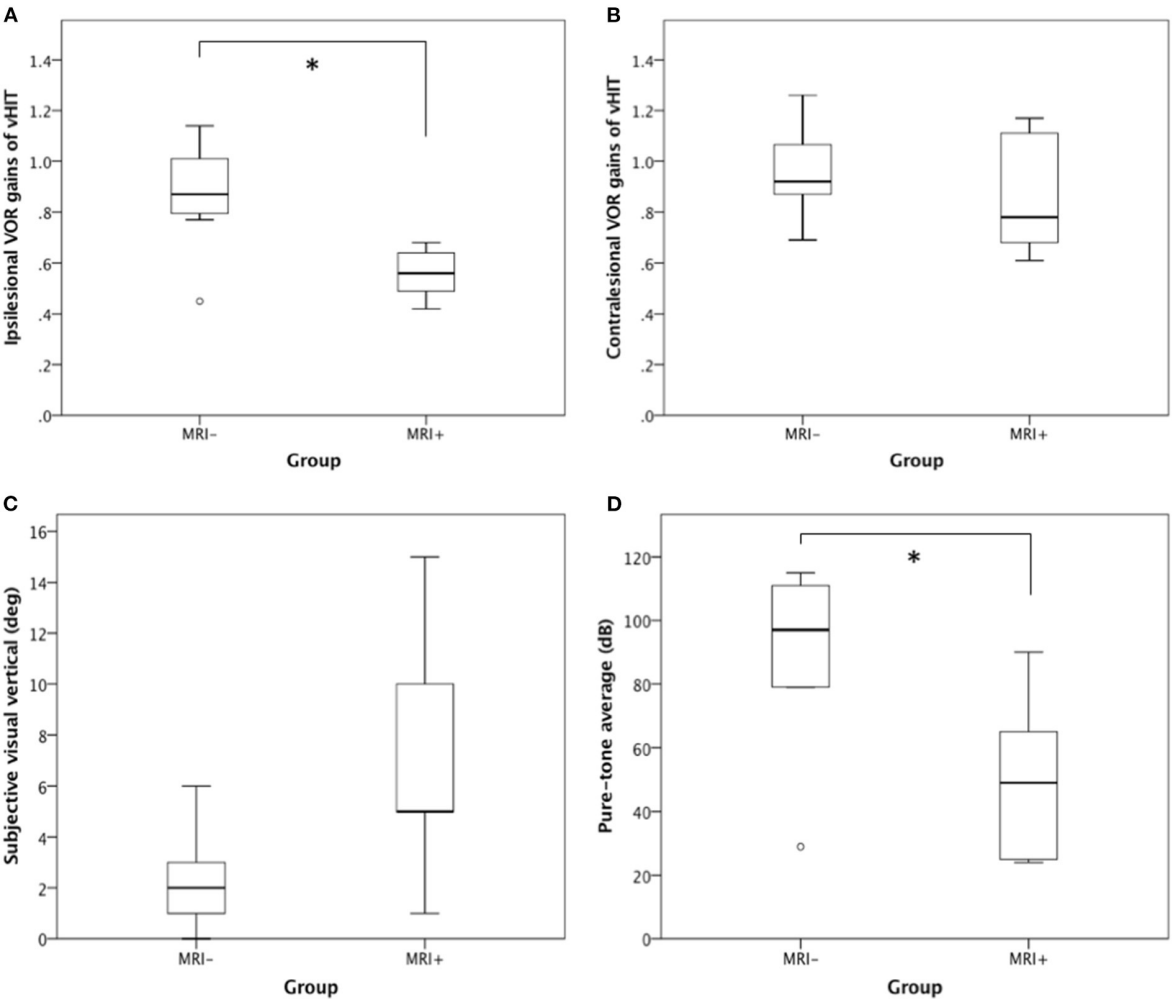
H-vHIT, SVV, and PTA between MRI+ and MRI– group. When compared with the MRI– group, the MRI+ group had **(A)** lower ipsilesional VOR gain in H-vHIT (0.56 ± 0.11 vs. 0.87 ± 0.24, *p* = 0.03), **(B)** similar contralesional VOR gain in H-vHIT (0.87 ± 0.25 vs. 0.96 ± 0.19, *p* = 0.43), **(C)** suggestion of greater SVV deviation (7.2 ± 5.4° vs. 2.3 ± 2.0°, *p* = 0.11), and **(D)** lesser degree of hearing loss on PTA (50.6 ± 28.0 dB vs. 89.8 ± 28.5 dB, *p* = 0.03). *Signifies statistically significant difference.

We compared the VOR gains of H-vHIT between the two groups. The MRI+ group had significantly lower ipsilesional VOR gain than the MRI– group (0.56 ± 0.11 vs 0.87 ± 0.24, *p* = 0.03) (Figure 2A). The contralesional VOR gains did not show significant difference between MRI+ and MRI– groups (0.87 ± 0.25 vs 0.96 ± 0.19, *p* = 0.43) (Figure 2B).

### Subjective visual vertical

All but one (in MRI– group) patients underwent testing for shift of the subjective visual vertical (using the bucket test) 2–3 days after vertigo onset. Six patients (4/5 in MRI+ and 2/7 in MRI–) has SVV deviations toward the ipsilesional side while one MRI– patient (1/7) deviated toward the contralesional side. When comparing the absolute values of SVV, the MRI+ group tended to have greater SVV deviation than MRI– though the difference did not reach statistical significance (mean deviation 7.2° in MRI+ vs 2.3° in MRI–, *p* = 0.11) (Figure 2C).

### Pure-tone audiometry

All patients underwent pure-tone audiometry 3–7 days after symptom onset. Patients in the MRI+ group had lesser degree of sensorineural hearing loss than those in the MRI– group (PTA 50.6 ± 28.0 in MRI+ vs 89.8 ± 28.5 in MRI–, *p* = 0.03) (Figure 2D). Seven MRI– patients (7/8) met the formal definition of SSNHL while only two MRI+ patients (2/5) met SSNHL. Moreover, two MRI+ patients had regained normal hearing when undergoing audiometry 5–7 days after onset.

## Discussion

In this study, we consecutively reviewed inpatients with AVS associated with acute unilateral hearing loss, to determine whether bedside neuro-otologic testing could differentiate central from peripheral lesions.

The study has two important findings. First, unlike in AVS without hearing loss, in AVS with hearing loss positive H-HIT is not a benign sign predicting a peripheral lesion. In fact, in the present study, positive H-HIT is more strongly associated with lateral pontine lesions (e.g., AICA infarction) than is negative H-HIT. Second, contrary to traditional teaching, the vestibular signs of the MRI+ group commonly mimics peripheral vestibulopathy while the presentation of MRI– group can be confused with central vestibulopathy given some combination of negative H-HIT, atypical positional nystagmus, or paucity of nystagmus in many MRI negative cases.

The MRI+ group in our study presented with the vestibular signs of unilateral peripheral vestibulopathy: ipsilesional vestibular loss on H-HIT, spontaneous nystagmus beating to contralesional side, and fixed-direction positional nystagmus. Clinical signs suggesting a central lesion, such as direction-changing GEN and skew deviation (18, 19), occurred in fewer than half of MRI+ patients. In other words, more than half of patients with central lesions have HINTS exam findings that point to a peripheral etiology (Table 2). As such, this group is at high risk of misdiagnosis. Our study therefore supports the use of acute hearing loss as a red flag feature in AVS to ensure that an acute lateral pontine lesion, such as an AICA stroke, is not missed. In other words, our study supports the use of HINTS Plus (where the finding of acute hearing loss prompts neuroimaging to search for a central cause).

**Table 2:** The distribution of the 13 patients in HINTS battery and MRI findings.

	**MRI+**	**MRI–**
Central HINTS	Pt #2, #3 (*n* = 2)	Pt #7, #8, #9, #10, #11 (*n* = 5)
Peripheral HINTS	Pt #4, #5, #6 (*n* = 3)	Pt #1, #12, #13 (*n* = 3)
Total, *n*	5	8

Central HINTS: negative head impulse test OR direction-changing nystagmus OR positive test of skew.

Peripheral HINTS: positive head impulse test AND unidirectional horizontal nystagmus AND negative test of skew.

MRI+: with posterior fossa lesions on MRI.

MRI–: without posterior fossa lesions on MRI.

Understanding the test characteristics of the three items of HINTS may partially explain our findings. Among the three items, direction-changing nystagmus and skew deviation are highly specific to central lesions (92 & 98%) but their sensitivities are low (38 & 30%). The high sensitivity for HINTS to identify central lesions mainly comes from the contribution of H-HIT (sensitivity 85%) (20). However, when applied in our specific condition—AVS plus hearing loss—the positive H-HIT no longer indicates a pure peripheral lesion. In fact, it may even suggest proximal AICA infarction. Thus, the sensitivity of H-HIT decreases in this situation and cannot be well-compensated by the other two tests, which in turns limits the applicability of HINTS for AVS plus hearing loss, and necessitates the more conservative approach (HINTS Plus).

Although positive H-HIT is usually a peripheral sign, the literature has shown several exceptions. Lesions in multiple brain structures can cause positive HIT, including in CN VIII fascicles in the brainstem, the vestibular nucleus, the cerebellar flocculus, and the nucleus prepositus hypoglossi (21–23). Most notably, a positive H-HIT is often seen in AICA stroke because the labyrinthine artery originates from AICA (24). The presence of lateral pontine infarction with AVS and hearing loss implies the vessel occlusion occurs at a proximal site of AICA, which in turns results in whole labyrinthine infarction and typical presentation of unilateral vestibular loss. Interestingly, in a previous study some patients with AICA stroke have bilateral vestibular hypofunction in vHIT (24). In our study, this finding was also seen in two cases of AICA stroke (Patients #4 & #5) and one with a lateral pontine demyelinating lesion (patient #3) (25). It cannot be explained by labyrinthine infarction. On the other hand, the cerebellar flocculus has been shown to inhibit low-frequency VOR but facilitate high-frequency VOR (23). Impairment of bilateral high-frequency VOR gains in unilateral floccular infarction has been proven not only by vHIT but also by the HIT using scleral search coils (23, 26). Since the flocculus belongs to AICA territory, in some cases of AICA stroke, the bilateral hypofunction in vHIT testing may be caused by floccular ischemia. Neural substrates in the lateral pons such as the vestibular commissure may equally be responsible for this phenomenon.

The peripheral AVS with hearing loss, usually termed “labyrinthitis,” is expected to have typical signs of unilateral vestibular loss. Surprisingly, in our study, fewer than half of the MRI– patients had positive H-HIT. Furthermore, three patients with normal H-HIT and weak nystagmus presented with isolated PC hypofunction. This subgroup is against the rule of HINTS and may be misdiagnosed with central vestibulopathy. Indeed, this phenomenon has been reported in other studies (27–29). In one study with 29 SSNHL patients 45% had vestibular dysfunction, in which 53% showed isolated PC impairment (28). Another study showed the same finding in 30% of 27 patients with acute vertigo and SSNHL (27). The most likely reason behind this phenomenon is infarction of the common cochlea artery, which supplies the cochlea and PC (27, 28, 30). In other words, this finding supports the vascular etiology of SSNHL (i.e., occlusion of AICA terminal branch). Clinicians should be aware of the possibility of distal vascular occlusion in patients with AVS plus hearing loss even if MRI brain fails to show a stroke. Critical control of vascular risk factors and even antithrombotic agents should be considered to prevent future stroke even in cases where the MRI is negative.

In our study, there were four other MRI– patients presenting with persistent geotropic or apogeotropic nystagmus during supine-roll test. Of course, the most common etiology of geotropic or apogeotropic nystagmus is H-BPPV. In our cases, however, the persistence of geotropic nystagmus did not match H-BPPV, and the apogeotropic nystagmus was not eliminated by the repositioning maneuver for H-cupulolithiasis (31). Instead, they corresponded to the previously reported “light cupula” and “heavy cupula,” which is probably due to change of specific gravity in the cupula or endolymph, resulting in cupula deviation to one side during head position change (32–34). The mechanism of so-called light/heavy cupula syndrome is thought to be either vascular (33–35) or inflammatory (33) in origin, and is sometimes associated with unilateral hearing loss (34). Our study supports previous findings, and further confirms that light/heavy cupula syndrome can be associated with SSNHL. More importantly, the H-HIT findings in these patients were not consistent, in which two patients had positive H-bHIT but negative H-vHIT, one had negative H-bHIT and positive H-vHIT, and one had both negative H-bHIT and H-vHIT. To our knowledge, apogeotropic or geotropic nystagmus can result from central lesions involving nodulus, uvula, or middle or inferior cerebellar peduncle (36–38). Accordingly, the vestibular signs in this subgroup are also inconsistent with those typical signs in unilateral peripheral vestibulopathy, and easily confused with central positional nystagmus.

In our study, audiograms showed the degree of hearing loss was less severe in the MRI+ group than the MRI– group, and two MRI+ patients with initial hearing loss had recovered during audiometry testing. This is a counterintuitive finding because hearing loss caused by vascular occlusion is theoretically more profound and irreversible than other causes (39).

In our study, the MRI+ group showed a tendency toward greater SVV deviation, which may hint at a role of SVV in identifying central lesions. Though three MRI+ patients had other focal neurological signs, two presented with peripheral-type facial palsy and one was initially misdiagnosed with Ramsay Hunt syndrome.

Our study has several limitations. First, our study is retrospective and sample size is small. A prospective study with larger sample size is warranted in the future to validate our findings. Second, we did not routinely perform vertical-canal vHIT, but only performed it when H-vHIT was normal. Third, we did not perform vestibular evoked myogenic potentials. Last, due to logistical factors at our center, our cases underwent audiometry 3–7 days after symptom onset so quantitative results of hearing loss in the first 2 days was not available.

In conclusion, in this study of 13 patients with AVS plus hearing loss, five patients (38.5%) had an acute lateral pontine lesion. All five of these patients had H-HIT that pointed incorrectly toward a peripheral etiology. While the other two items in the HINTS battery (direction-changing nystagmus and skew deviation) would correctly have identified two additional cases of stroke, and one patient had other focal findings (facial palsy), two of the five patients had no central findings (Table 2). This put them at risk of misdiagnosis. These findings suggest that every patient with AVS plus hearing loss should undergo brain MRI, and clinicians should be aware of the possibility of a lateral pontine lesion when a positive H-HIT is seen in patients with AVS with hearing loss.

## Data availability statement

The raw data supporting the conclusions of this article will be made available by the authors, without undue reservation.

## Ethics statement

The studies involving human participants were reviewed and approved by Research Ethics Committee of Taichung Tzu Chi Hospital. Written informed consent for participation was not required for this retrospective study.

## Author contributions

AB is responsible for interpretation of the data and drafting of the manuscript. TPC is responsible for collection, analysis, interpretation of the data, and drafting of the manuscript. All authors contributed to the article and approved the submitted version.

## Conflict of interest

The authors declare that the research was conducted in the absence of any commercial or financial relationships that could be construed as a potential conflict of interest.

## Publisher's note

All claims expressed in this article are solely those of the authors and do not necessarily represent those of their affiliated organizations, or those of the publisher, the editors and the reviewers. Any product that may be evaluated in this article, or claim that may be made by its manufacturer, is not guaranteed or endorsed by the publisher.
